# Approaching Protein Barriers: Emerging Mechanisms of Replication Pausing in Eukaryotes

**DOI:** 10.3389/fcell.2021.672510

**Published:** 2021-05-28

**Authors:** Maksym Shyian, David Shore

**Affiliations:** Department of Molecular Biology, Institute of Genetics and Genomics of Geneva (iGE3), University of Geneva, Geneva, Switzerland

**Keywords:** fork pausing complex, replication fork, replication fork barrier (RFB), replication fork slowdown, MTC (Mrc1-Tof1-Csm3), topoisomerase I (Top1), topoisomerase II (Top2)

## Abstract

During nuclear DNA replication multiprotein replisome machines have to jointly traverse and duplicate the total length of each chromosome during each cell cycle. At certain genomic locations replisomes encounter tight DNA-protein complexes and slow down. This fork pausing is an active process involving recognition of a protein barrier by the approaching replisome *via* an evolutionarily conserved Fork Pausing/Protection Complex (FPC). Action of the FPC protects forks from collapse at both programmed and accidental protein barriers, thus promoting genome integrity. In addition, FPC stimulates the DNA replication checkpoint and regulates topological transitions near the replication fork. Eukaryotic cells have been proposed to employ physiological programmed fork pausing for various purposes, such as maintaining copy number at repetitive loci, precluding replication-transcription encounters, regulating kinetochore assembly, or controlling gene conversion events during mating-type switching. Here we review the growing number of approaches used to study replication pausing *in vivo* and *in vitro* as well as the characterization of additional factors recently reported to modulate fork pausing in different systems. Specifically, we focus on the positive role of topoisomerases in fork pausing. We describe a model where replisome progression is inherently cautious, which ensures general preservation of fork stability and genome integrity but can also carry out specialized functions at certain loci. Furthermore, we highlight classical and novel outstanding questions in the field and propose venues for addressing them. Given how little is known about replisome pausing at protein barriers in human cells more studies are required to address how conserved these mechanisms are.

## Introduction

In order to duplicate chromosomes, replicative polymerases have to access each base in the DNA. This requires removing DNA-binding proteins, resolving topological constraints and melting the DNA double helix step-by-step along the whole length of each chromosome. A chromosome’s features vary along its length. Accordingly, it is not surprising that replication elongation rates are variable and that forks tend to slow down at regions where the bases are difficult to access, due to DNA secondary structures [resulting, for example, from trinucleotide and inverted repeats (Voineagu et al., 2008, 2009)], base modifications (including covalent protein binding), excess superhelical tension [such as at termination zones generated by converging forks (Fachinetti et al., 2010)], or the tight binding of proteins or protein complexes. In this review we focus on non-covalent proteinaceous replication fork barriers (RFBs) and primarily refer to studies of the budding yeast *Saccharomyces cerevisiae*, where the understanding of DNA replication mechanisms is most complete. We discuss pausing at RFBs from various perspectives: detection methods, diversity, regulators, proposed physiological roles, and finish by summarizing emerging models and outstanding questions in the field.

### Bumps Along the Road: The Rate of Replisome Movement Varies Across the Genome

**FIGURE 1:**
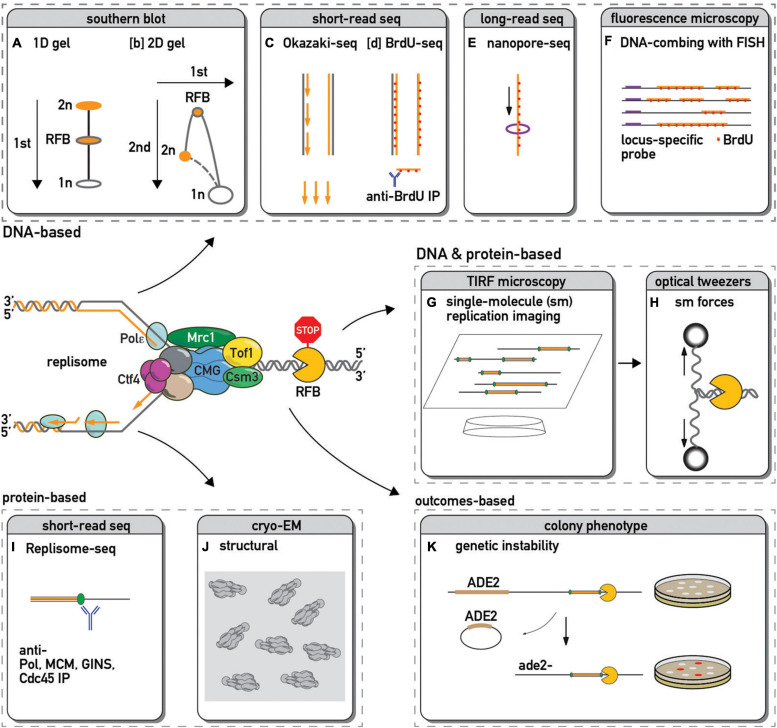
Methods used to study replication fork barriers and replisome pausing. The DNA in and around a paused replication fork is often detected by: 1-dimensional [1D, **(A)**–(Kobayashi et al., 2004)] or 2-dimensional [2D, **(B)**–(Brewer and Fangman, 1988)] gel electrophoresis followed by Southern blot and hybridization with a probe specific to a locus of interest; by sequencing and genome mapping of Okazaki fragments [**(C)**–(McGuffee et al., 2013)] or immunoprecipitated pieces of sonicated DNA that have incorporated a modified nucleotide analog such as BrdU [**(D)**–(Peace et al., 2014)]; by sequencing long stretches of DNA using nanopore technology to infer edges of BrdU incorporation and hence fork positions [**(E)**–(Muller et al., 2019)]; by microscopic examination of DNA fibers stretched on slides and immunostained with fluorescently labeled antibody against BrdU or other analogs [**(F)**–(Pasero et al., 2002)]. This fluorescent *in situ* hybridization (FISH) approach with a locus-specific probes allow one to focus only on DNA in the vicinity of a specific barrier. Single-molecule fluorescence microscopy methods allow real-time visualization of replication [**(G)**–(Sparks et al., 2019)] or even additional manipulation of the process by changing forces applied to DNA next to a barrier [**(H)**–(Berghuis et al., 2018)]. The chromosomal locations of replisome protein components can be detected by chromatin immunoprecipitation (ChIP) followed by PCR or high throughput DNA sequencing [**(I)**–(Azvolinsky et al., 2009; Sekedat et al., 2010)], allowing one to infer sites of pausing for specific factors or complexes. Cryo-electron microscopy yields structural insights into replisome component positions at a barrier [**(J)**–(Baretic et al., 2020)]. Since pausing sites are associated with an increase in recombination, loss of a genetic marker next to a barrier might serve as a proxy of pausing efficiency [**(K)**–(Kaeberlein et al., 1999)].

The pioneering work of Brewer and Fangman (1988), who developed a 2-dimensional gel electrophoresis (2D gel) Southern blot method to resolve and quantify replication intermediates, allowed the first, albeit indirect measure of relative replisome velocity at specific genomic loci (see Figure 1 and below). Their study, which focused on the rDNA repeat locus in yeast, was the first of many to show that DNA replication fork speed appears to decrease dramatically at certain sites, a phenomenon referred to interchangeably as: “pausing,” “slowdown,” “arrest,” or “stalling.” These definitions contrast with fork “collapse,” which is defined as an irreversible event involving DNA breakage at the fork and replisome dissociation from the template, though the latter outcome is controversial (De Piccoli et al., 2012).

In contrast to site-specific replisome pausing, certain chemical and genetic manipulations lead to a generally uniform change in fork speed. For example, general fork slowdown is caused by dNTP depletion following treatment with the ribonucleotide reductase inhibitor hydroxyurea [HU; (Alvino et al., 2007)], replicative polymerase inhibition by aphidicolin treatment (Pacek et al., 2006), and by loss of *MRC1* gene function in yeast (Hodgson et al., 2007) or of *TIMELESS* in mammalian cells (Somyajit et al., 2017). Somewhat surprisingly, other chemical or genetic perturbations lead to global fork acceleration, such as a decrease in the number of activated origins (Zhong et al., 2013), cohesin acetylation (Terret et al., 2009), PARP inhibition (Maya-Mendoza et al., 2018), or loss of peroxiredoxin 2 (PRDX2), a detector of reactive oxygen species [ROS; (Somyajit et al., 2017)].

Some of these general effects on fork movement may also be associated with changes in local fork rates at barriers. For instance, while causing global replication deceleration (Alvino et al., 2007), HU paradoxically leads to decreased pausing at some protein RFBs (Krings and Bastia, 2004; Anand et al., 2012) through a still unknown mechanism. Of note, there is an inverse connection between global fork speeds and the frequency of origin firing (percent of potential origins that actually fire), where higher origin firing rates lead to slower replication elongation, most likely due to depletion of essential factors such as dNTPs (Zhong et al., 2013). Severe checkpoint mutants that cause unscheduled origin firing, such as *mec1*, *rad53*, and *mrc1*, have slower fork rates than mutants with less severe defects [e.g., *tof1*; (Hodgson et al., 2007; Crabbe et al., 2010)]. However, these mutants with the slowest fork rates are still able to slow down the replisome at protein barriers, while the more modestly affected *tof1* mutant is not (Calzada et al., 2005; Tourriere et al., 2005; Hodgson et al., 2007), as further discussed below. At another extreme, *cdc7* mutants, which fire fewer origins and thus have faster forks (Zhong et al., 2013), turn out to be deficient for local slowdown at barriers (Bastia et al., 2016). Thus, there is no simple rule relating global and local fork speeds and the two phenomena appear to be largely independent.

### Approaches to Study Fork Progression

Since the initial detection of fork pausing at a specific site in the yeast rDNA repeat by 2D gel analysis of replication intermediates (Brewer and Fangman, 1988) a large number of orthogonal methods have been developed to measure this phenomenon (some of which are depicted in Figure 1). These methods enable one to monitor aspects of either replication fork or replisome progression in cells, extracts, or reconstituted systems and report on the features of local and global replication pausing.

Broadly speaking, methods to detect pausing can be divided into two categories. The first of these quantifies DNA signatures, such as relative abundance of replicative structures (Brewer and Fangman, 1988) or nascent DNA at a replication fork (Peace et al., 2016). The second category of methods quantifies the abundance of protein components of the replisome [e.g., polymerase (Azvolinsky et al., 2009) or helicase (Sekedat et al., 2010)], either at specific regions or genome-wide, under the assumption, analogous to that used in 2D gel analysis, that variations reflect the time required by the replisome to traverse any given site. However, given the possibility of polymerase-helicase uncoupling (Katou et al., 2003; Pacek et al., 2006; Graham et al., 2017) one has to be cautious about inferring replisome position (a protein-based measure) from the fork position (DNA-based), and vice versa. Recent single-molecule replication (Sparks et al., 2019) and DNA unwinding (Berghuis et al., 2018) imaging methods allow for combined detection of protein and DNA chromatin components, and introduction of a labeled barrier [such as Cas9, (Vrtis et al., 2021)] helps one to focus on events around it. Along the same lines, the field would benefit from the development of new methods capable of simultaneous co-detection of replisome proteins and nascent fork DNA components at single-nucleotide resolution. Thus, the above list is a standard “menu” (albeit not exhaustive) of orthogonal methods from which to choose when addressing classical and emerging questions in replisome progression.

### The Chromosomal Landscape of Replication Barriers

In budding yeast, replisome pausing was first discovered, by 2D gel analysis (Figure 1), at a specific site adjacent to the unique replication origin in all rDNA repeats (Brewer and Fangman, 1988) and at tRNA genes (Deshpande and Newlon, 1996). In both cases, replication slowdown was initially believed to stem from replication-transcription collisions. However, it was later found that rDNA pausing is independent of transcription (Brewer et al., 1992) but instead requires a specific DNA-binding protein Fob1 (FOrk Blocking less 1 (Kobayashi and Horiuchi, 1996)). Similarly, pausing at tRNA genes was shown to require assembly of a transcription pre-initiation complex (Ivessa et al., 2003), but to operate independently of transcription itself (Yeung and Smith, 2020).

**FIGURE 2:**
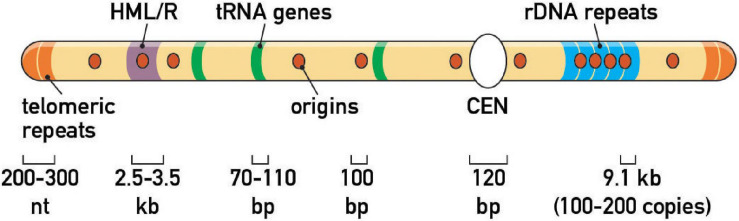
Endogenous fork barriers. Schematic representation of the repertoire of prominent replication fork barriers at a schematic budding yeast chromosome. Note that *HML*/*HMR* heterochromatic silencers and rDNA tandem repeats are on different chromosomes *in vivo* (chr III and XII, respectively). Average size of genomic features is also indicated.

Zakian and colleagues expanded this initial picture by screening candidate protein-DNA complexes for RFB activity, including centromeres (CEN), telomeres and inactive replication origins (e.g., those found at *HML* and *HMR* mating-type gene “silencer” elements). This targeted approach revealed an estimated total of ∼1,400 RFBs in the yeast genome (Ivessa et al., 2003; Figure 2). More recently, the establishment of inducible, ectopic RFBs [e.g., a Rtf1/Rtf2-mediated RTS1 RFB in fission yeast (Lambert et al., 2005), a Fob1-dependent eRFB in budding yeast (Bentsen et al., 2013; Krawczyk et al., 2014), a Tus/Ter-dependent RFB in mammalian cell lines (Willis et al., 2014), and LacI/LacO arrays in Xenopus egg extracts (Dewar and Walter, 2017) and mammalian cells (Ishimoto et al., 2021)] has laid the foundation for more detailed studies of the consequences of pausing on genome integrity, cell cycle progression, replication checkpoints, and chromosome segregation (see below).

Even more recently, nuclease-dead Cas9 (dCas9) was repurposed as a protein (or protein-covered R-loop) replisome barrier. dCas9 efficiently blocks the yeast replisome *in vivo* (Doi et al., 2021) and all replisomes tested *in vitro* [viral, bacterial and yeast; (Whinn et al., 2019)]. Future studies will likely utilize dCas9 barrier systems to glean more insights into pausing mechanisms.

Fork barriers are either polar (Fob1-rRFB, Tus/Ter) and stall replisomes advancing from one side only, or non-polar and stall replisomes arriving from either direction (e.g., CEN, tRNA, or *HML*/*HMR* silencers). Barriers also vary in their efficiency (% of blocked forks) and strength (time the replisome spends on the barrier), with CEN barriers being transient [dozens of seconds (Deshpande and Newlon, 1996)] and Fob1-rRFB being very efficient, and strong, and thus serving as a replication termination site (Brewer and Fangman, 1988). The strongest barriers become replication termination sites if the blocked fork remains arrested until a converging fork arrives from another side of the barrier to rescue it (Fachinetti et al., 2010). In summary, fork pausing in eukaryotes is neither a passive nor indiscriminate process but instead requires specific *trans*-acting regulatory factors operating in either an orientation-specific or bi-directional fashion.

### Factors Mediating Pausing at the Replisome

**FIGURE 3:**
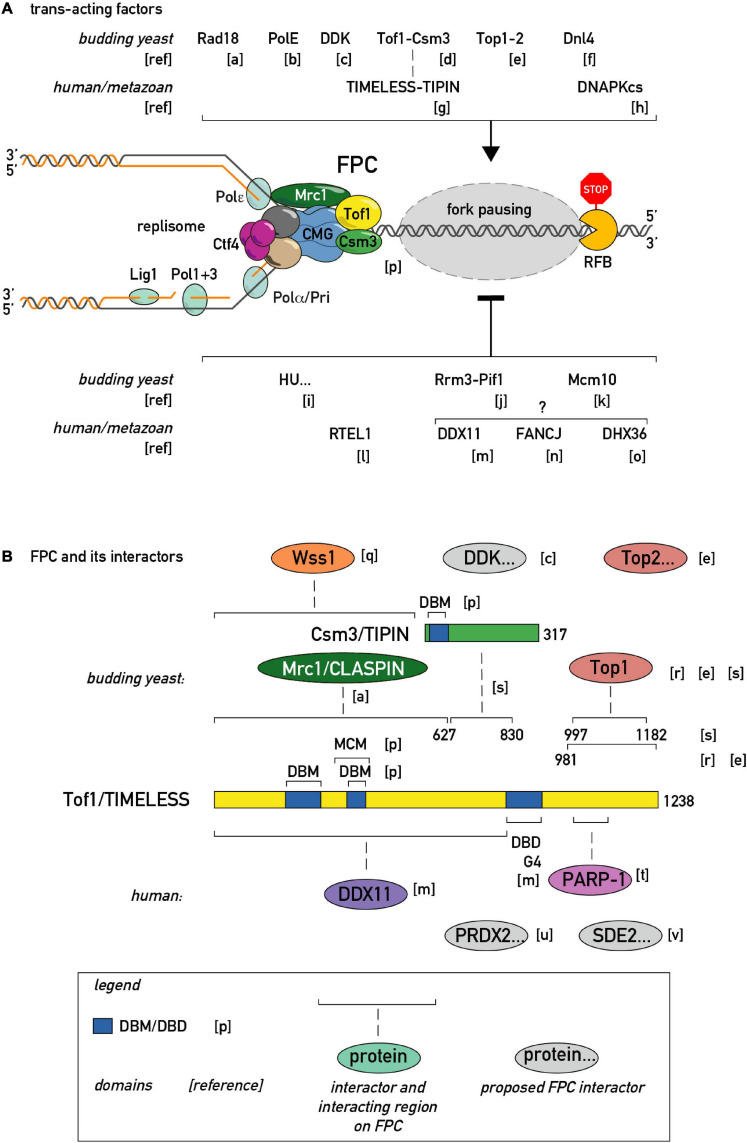
Regulators of fork pausing. **(A)** *Trans*-acting proteins and a chemical proposed to modulate replication fork progression through a barrier promoting pausing (top of the schematic with activatory arrow sign) or alleviating it (bottom of the schematic with inhibitory bar sign). **(B)** MTC (Mrc1-Tof1-Csm3) protein domain structure, proposed protein-protein interactions, and interacting regions in budding yeast and human cells. DBD/M—DNA binding domain/motif. MCM—MCM2-7 binding motif. G4—G-quadruplex binding region. [a]–(Yeung and Smith, 2020), [b]–(Hizume et al., 2018), [c]–(Bastia et al., 2016), [d]–(Mohanty et al., 2006), [e]–(Shyian et al., 2020; Yeung and Smith, 2020), [f]–(Fritsch et al., 2010), [g]–(Akamatsu and Kobayashi, 2015), [h]–(Janel-Bintz et al., 2020), [i]–(Anand et al., 2012), [j]–(Osmundson et al., 2017), [k]–(Langston and O’Donnell, 2017), [l]–(Sparks et al., 2019), [m]–(Lerner et al., 2020), [n]–(Schwab et al., 2013), [o]–(Sato et al., 2020), [p]–(Baretic et al., 2020), [q]–(O’Neill et al., 2004)., [r]–(Park and Sternglanz, 1999), [s]–(Westhorpe et al., 2020), [t]–(Xie et al., 2015), [u]–(Somyajit et al., 2017), [v]–(Rageul et al., 2020).

Replication pausing results from an interplay between a barrier of some sort and the replication machinery itself, broadly defined, and can be influenced by both positive and negative regulators acting directly at the replisome (Figure 3A). The first factors implicated in pausing were discovered in yeast genetic screens that scored for recombination (Figure 1), induced either by a short sequence from the rDNA repeats [in budding yeast; (Keil and McWilliams, 1993; Kobayashi and Horiuchi, 1996)] or during mating-type switching [in fission yeast; (Gutz and Schmidt, 1985)]. The budding yeast studies identified the *FOB1* gene, which encodes a DNA-binding protein required for pausing, and *RRM3*, which encodes a helicase, as a negative regulator of fork stalling. The fission yeast studies instead identified the *SWI1* and *SWI3* genes, both of which were shown to be pause-promoting factors.

#### Barriers to Fork Progression

Impediments to replication fork passage come in a multitude of forms, including DNA secondary structures (e.g., hairpins or G4 quadruplexes), covalent DNA modifications, including attached proteins, converging replication or transcription complexes, and tightly DNA-bound proteins or protein complexes. Despite these differences in the molecular nature of the various obstacles to replisome progression, a common set of replisome and replisome-associated factors are involved in regulating fork speed at these sites and in doing so helping to preserve genome integrity.

The first eukaryotic barrier protein to be identified, and perhaps the best studied to date, is the DNA-binding protein Fob1 (Figure 3A), which binds specifically to two sites within the rRFB and is absolutely required for fork pausing there. Fob1 is unusual amongst barrier proteins in that it is believed to act uniquely at the rDNA, possibly through a mechanism that involves wrapping of rRFB DNA around the protein itself (Kobayashi, 2003). Fob1 blocks fork progression in a polar manner, as do bacterial Tus proteins involved in replication termination (Elshenawy et al., 2015; Berghuis et al., 2018), through a mechanism still not clearly defined. Interestingly, in the distantly related fission yeast *S. pombe* the Fob1 ortholog is a protein called Sap1, which, unlike Fob1, is essential for viability and has multiple functions elsewhere in the genome, acting both in replication initiation (Guan et al., 2017) and as a general regulator of transcription (Tsankov et al., 2011). In addition, Sap1 participates in a specialized replication pausing event associated with the regulation of mating-type gene switching, as discussed below. Finally, replication pausing at the *S. pombe* rDNA also relies upon the transcription termination factor Reb1, whose mammalian homolog, TTF1, appears to act as the unique barrier protein.

As pointed out above, an additional set of prominent chromosomal features in budding yeast, including centromeres, telomeres, origins of replication, tRNA genes, and the two silent mating-type loci are site of fork pausing in yeast. All of these regions are characterized by proteins or protein complexes that bind specifically and often with high affinity to sequence motifs within them. Nevertheless, the precise nature of the barrier in these cases is less clear than at the rDNA. Telomeres constitute an interesting case where it is unclear *a priori* whether the barrier is due to a DNA structure (G4 quadruplexes that can form on telomeric TG-repeat sequences) or to the proteins that bind tightly to these sequences (see below).

Many barriers or obstacles to replication fork progression are “accidental” in nature, including DNA secondary structures, covalent DNA modifications and transcription complexes that can collide with opposing replisomes. For a more detailed discussion of these types of blocks, their resolution and consequences for genome stability we refer the reader to a series of excellent reviews (Mirkin and Mirkin, 2007; Lambert and Carr, 2013; Bastia and Zaman, 2014; Gadaleta and Noguchi, 2017; Stingele et al., 2017; Hizume and Araki, 2019).

#### Accessory Helicases Displace Barriers

Rrm3 and its paralog Pif1 are “accessory” replicative helicases that translocate in a 5′-to-3′ direction, opposite to that of CMG, the main replicative helicase [reviewed in Sabouri (2017)]. Both accessory helicases are believed to operate on the lagging strand template to actively assist CMG helicase at most barriers, including those at replication termination zones. Indeed, loss of Rrm3 and Pif1 has an additive effect on pausing at tRNA genes (Osmundson et al., 2017; Tran et al., 2017). Accordingly, recombinant Pif1 supports polymerase and helicase-polymerase complex progression through barriers *in vitro* (Schauer et al., 2020; Sparks et al., 2020). Budding yeast Pif1 also promotes fork progression through G-quadruplex (G4) DNA structures (Paeschke et al., 2011) and migrating D-loops formed during break induced replication [BIR; (Wilson et al., 2013; Chung, 2014; Liu et al., 2021)]. *In vitro*, Pif1 has been shown to promote bypass of dCas9, suggesting that it may act in general to remove both protein and R-loop blocks to replisome progression (Schauer et al., 2020). At the Fob1-rRFB, however, Rrm3 and Pif1 have confounding effects: whilst Rrm3 decreases pausing, as expected, Pif1 appears to have an unexplained opposite effect. The fission yeast *S. pombe* has only one Pif1/Rrm3 ortholog, Pfh1, which, similarly to Rrm3, promotes fork progression through various impediments.

Several 5′-3′ accessory helicases are candidates to fulfill the roles of yeast Rrm3 and Pif1 at metazoan replisomes, including RTEL1 (Vannier et al., 2013), DDX11 (Lerner et al., 2020), FANCJ (Sato et al., 2020), and DHX36 (Sato et al., 2020). RTEL1 was recently reported to assist replisome progression through non-covalent and covalent barriers (Sparks et al., 2019), while all these four helicases were implicated in promoting progression past G4 structures, reminiscent of Pif1’s role in yeast. It will be of interest to test whether *in vivo* progression through G4 structures is problematic due to DNA structure alone or due to an (additional) effect of specific G4-binding proteins.

It is worth noting that the question of what happens to barrier-forming proteins during and just after replication fork passage has hardly been addressed. Is the barrier protein displaced temporarily/terminally or does the fork complex enigmatically “jump” over it, as was proposed to happen in the context of covalent DNA-protein crosslink (DPC) bypass (Sparks et al., 2019)? Do displaced proteins immediately re-bind following replisome bypass? If the barrier protein re-binds DNA in the wake of the helicase, how fast does it do so? Is there sufficient time for polymerases to synthesize nascent DNA or does the re-bound barrier protein preclude further polymerase(s) action? Is there a pathway for chromatid specific RFB segregation or does the barrier re-form randomly on either sister chromatid? Are barrier proteins post-translationally modified, unfolded, or degraded during pausing and bypass? Is there a way for a cell to distinguish non-covalent tight DNA complexes and covalent DPCs or do DPC proteolytic pathways (Stingele et al., 2017) also operate on tight protein barriers? It is worth noting in this regard that yeast fork protection factor Tof1 (see below) was reported to interact with the DPC protease Wss1 (O’Neill et al., 2004), though the physiological relevance of this interaction remains to be determined.

#### Pause-Promoting Factors Slow Down Forks

The list of regulators that enhance pausing is also expanding (Figures 3A,B). At the level of the replisome itself, an evolutionary conserved heterodimeric complex consisting of Tof1 and Csm3 in budding yeast and Swi1 and Swi3 in fission yeast, dubbed the Fork Pausing/Protection Complex (FPC), has been shown to play a primary role in replisome pausing (Noguchi et al., 2004; Mohanty et al., 2006). Given the absence of known catalytic activities in FPC components, the first model for FPC activity postulated that it inhibits the Rrm3 “sweepase” activity that removes barriers (Mohanty et al., 2006). Later it was clarified that Rrm3 and FPC act for the most part independently of each other, since the FPC is still required for wild type pausing levels in cells devoid of Rrm3 (Torres et al., 2004; Shyian et al., 2020). Thus, pausing at all physiological endogenous proteinaceous RFBs studied so far in budding yeast is inhibited by action of the Rrm3 helicase and promoted by the FPC. Significantly, a recent structure of CMG helicase engaged with the FPC and fork DNA revealed that the FPC is situated in front of the helicase, and extensively interacts with the CMG itself and with incoming DNA (Baretic et al., 2020). However, a single-molecule study showed that the MTC complex interaction with CMG is dynamic, that is, prone to dissociation/reassociation reactions (Lewis et al., 2017). It will thus be of great importance to investigate if the MTC-CMG interaction is also dynamic *in vivo*, and if so, whether this process is regulated and of functional significance.

It appears that most positive pausing regulators *in vivo* channel in some way through the FPC complex (Figures 3A,B). Although the FPC component Tof1 was initially identified as a topoisomerase I (Top1)-interacting protein, it is only very recently that Top1 (and Top2) were identified as essential for fork pausing at rRNA and tRNA RFBs [see below; (Shyian et al., 2020; Yeung and Smith, 2020)]. In addition to topoisomerases, the list of FPC interactors in different model organisms is growing (Figure 3B). Some of these were shown to act in the fork pausing pathway. For example, the Dbf4-dependent kinase (DDK), required for replication origin firing, was proposed to regulate the Tof1-CMG interaction (Bastia et al., 2016). It is worth noting that DDK is recruited to the replisome by FPC in pre-meiotic replication (Murakami and Keeney, 2014) raising the question of whether FPC may employ DDK to modulate fork speed. The recently identified mammalian TIMELESS interactor, PRDX2, was implicated in fork speed modulation (Somyajit et al., 2017), giving another precedent for an FPC interactor adjusting fork rates. Along these lines, it will be interesting to test whether PARP-dependent fork speed regulation (Maya-Mendoza et al., 2018) is channeled through FPC, given the known TIMELESS-PARP interaction (Xie et al., 2015).

At this point it is worth noting that the FPC (Tof1/TIMELESS-Csm3/TIPIN) and its partner Mrc1/CLASPIN also carry out other functions. For example, they are also required for proper DNA replication checkpoint function (Foss, 2001; Katou et al., 2003; Noguchi et al., 2003). Surprisingly, though, DNA replication and damage checkpoints are not essential for pausing (Calzada et al., 2005). Accordingly, fork pausing and replication checkpoint signaling functions were recently reported to be separable within the FPC complex itself (Shyian et al., 2020; Westhorpe et al., 2020). The FPC is also involved in sister chromatid cohesion establishment in a pathway shared with Ctf4 and Chl1 (Xu et al., 2007). Interestingly, mammalian TIMELESS interacts with the Chl1 ortholog DDX11 to cooperate in G4 bypass (Lerner et al., 2020). It is unknown, however, if DDX11 (or Chl1) assists replisome progression through proteinaceous RFBs. It will be interesting to determine if the FPC’s role in cohesion is related to its role in replisome speed control, or if these functions are independent and perhaps genetically separable.

Recent *in vitro* studies showed that a core replisome composed of CMG-FPC and polymerases is able to confer some degree of pausing even on a linear template and in the absence of many of the other factors listed on Figure 3A and required *in vivo* (Hizume et al., 2018; Baretic et al., 2020). It is evident, however, that *in vivo* pausing at chromatinized, topologically-constrained substrates requires not only the “core” FPC but also additional factors, such as Top1/2, Rad18 (Yeung and Smith, 2020) and others (Figure 3A). It will be of particular interest to build further upon existing *in vitro* systems and reveal the minimal set of factors required to reconstitute *in vivo*-like pausing efficiency and the interplay of various pausing regulators.

Some observations point toward FPC-independent pausing mechanisms. For instance, a non-catalytic replisome component, Mcm10, was reported to be required for FPC-independent lagging strand barrier bypass in an *in vitro* study (Langston and O’Donnell, 2017). Another set of studies showed that treating budding and fission yeast with the replication stress agent HU leads to a loss of pausing at some RFBs (Krings and Bastia, 2004; Anand et al., 2012) through an unknown mechanism that was proposed to be independent of canonical FPC-dependent pausing (Anand et al., 2012). Furthermore, pausing at artificially engineered Tus/Ter barriers in yeast is Tof1-independent and also unaffected by Rrm3 (Larsen et al., 2014), perhaps reflecting the highly mechanical nature of this particular barrier (Berghuis et al., 2018).

One important feature of the list of pausing regulators depicted on Figure 3A is that it is currently unclear if all these factors are continuously present on the fork or are specifically recruited/evicted in the vicinity of a barrier. Along the same line, it is unknown whether a different set of accessory factors is recruited/evicted when the replisome approaches RFBs of a different nature. Are these factors removed after having done their job or do they persist at the replisome and thus carry a “memory” of progression through a barrier? Indeed, recent studies in fission yeast suggest that paused forks may be restarted by homologous recombination and have different properties than the canonical replisome (Naiman et al., 2021).

Whether constitutively present or transiently recruited, pausing regulators might be expected to be tightly regulated themselves (as the saying goes, who watches the watchmen?). Indeed, one can imagine that pausing becomes deleterious in cells experiencing severe under-replication due to genotoxic stress and that mechanisms reversing pausing in these conditions may be necessary for complete genome replication. In line with this possibility, replication stress induced by HU relieves pausing in both fission (Krings and Bastia, 2004) and budding (Anand et al., 2012) yeasts. However, it is still unknown how HU elicits this effect, whether it is mediated by canonical DNA replication checkpoint (DRC) or DNA damage repair pathways, or whether the FPC is the target of this regulation. Similarly, it is unknown whether pausing is regulated during S phase, or under replication stress, or in cells experiencing DNA damage.

Thus, *in vivo* replisome pausing detected at an RFB is a complex function of the blocking protein, both positive (e.g., FPC) and negative (accessory helicase) modulators, and possibly additional levels of regulation acting on these different players.

### DNA Topology, Topoisomerases, and Replisome Pausing

The intertwining of the two DNA strands once every ∼10 base pairs implies the existence of a robust mechanism to separate them during replication (Watson and Crick, 1953). The discovery of abundant topoisomerase enzymes in both prokaryotes and eukaryotes provided a plausible scheme to resolve this problem [for a perspective see Wang (2002); reviewed more recently in Baxter (2015); Keszthelyi et al. (2016)]. This was followed by pioneering genetic studies in yeast which demonstrated that Top1 and Top2 act redundantly as a “swivel” required for DNA replication in this model eukaryotic system (Brill et al., 1987). Together with other studies this work led to the view that Top1 primarily acts ahead of the fork to relieve positive supercoiling, but can be substituted for by Top2, which has the unique ability to act behind the fork to resolve sister chromatid intertwines (Baxter, 2015; Schalbetter et al., 2015).

Intriguingly, Top1 was shown to interact, in a yeast two-hybrid screen, with Tof1 [Top1-interacting factor 1; (Park and Sternglanz, 1999)], which was later shown to be a component of the FPC, as described above. However, Tof1, together with Csm3, had been proposed act in pausing as negative regulators of the Rrm3 helicase, which itself was thought to act directly to overcome fork blocks [(Mohanty et al., 2006), reviewed in Gadaleta and Noguchi (2017); Lawrimore and Bloom (2019)]. Furthermore, the action of Top1 in front of a replication fork might be expected to promote fork progression, rather than favoring pausing, as does the FPC. Indeed, in a highly purified *in vitro* replication system Tof1/Csm3 are required to achieve *in vivo* rates of synthesis (Yeeles et al., 2017). The possible significance of the Top1-Tof1 interaction in replication pausing was thus largely overlooked for many years. We recently revisited this problem and showed, as careful examination of earlier findings implied, that Tof1 (and Csm3) act in pausing in a manner largely independent of Rrm3 (Larcher and Pasero, 2020; Shyian et al., 2020).

**FIGURE 4:**
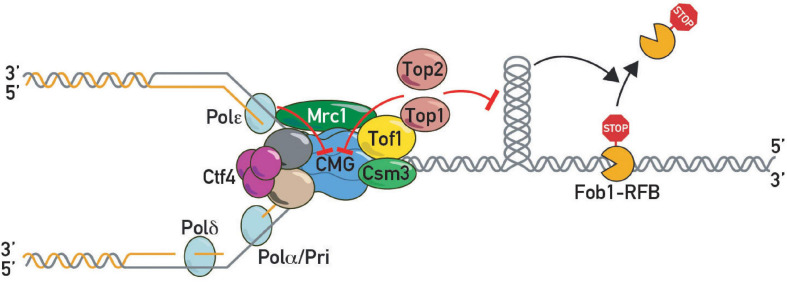
DNA topoisomerases in pausing. Topoisomerase I and II slow down replication forks at protein barriers either by direct inhibition of CMG helicase or indirectly by preventing build-up of barrier-disrupting DNA topology.

These findings suggested that as yet unidentified factors might act together with Tof1-Csm3 in fork pausing. In a search for such factors, we carried out a forward genetic screen using rDNA stability as a read-out. This screen led to the identification of a hypomorphic allele of *TOP1* (Shyian et al., 2020). Subsequent work demonstrated that Tof1 recruits Top1 to the replication fork through its C-terminal domain, where it acts redundantly with Top2 to promote fork pausing (Shyian et al., 2020). This function of Tof1 is genetically separable from its role in DNA replication checkpoint activation. Concurrent studies from the Baxter laboratory (Westhorpe et al., 2020) are consistent with these findings and provide a more detailed molecular dissection of the multiple roles of Tof1 in the control of fork pausing, checkpoint activation, fork stabilization and polymerase coupling. On the basis of these and other findings a new model for pausing was proposed, called sTOP, for “slowing down with topoisomerases I-II,” in which a direct interaction between Tof1 and either Top1 or Top2 slows down the fork as it reaches a barrier and promotes replisome stability there (Figure 4). The underlying mechanisms remain obscure (see below). Moreover, it remains unclear how Top2 is recruited to the replisome, for example through as yet unknown protein-protein interactions or DNA topology. Recruitment of Top2 ahead of the fork may be favored by its biophysical preference for a single parental chromatid (Le et al., 2019).

The presence of topoisomerases in front of a replication fork poses a potential danger since their normally transient DNA cleavage intermediates can be trapped by various forms of DNA damage or drugs, which can cause replication fork run-off at the leading strand and generation of a DNA double-strand break (DSB; (Strumberg et al., 2000). This suggests that topoisomerase activity must be tightly coordinated with that of the replicative helicase. Our recent studies implicate the Tof1 C-terminus in this process, since *tof1-*ΔC mutants are sensitive (although less so than a *tof1*Δ mutant) to both camptothecin and etoposide, drugs that trap Top1 and Top2 cleavage complexes, respectively (Shyian et al., 2020). Interestingly, as judged from the additive effects of *tof1* and *mrc1*Δ mutants (Katou et al., 2003; Shyian et al., 2020), the protection against trapped topoisomerases conferred by the Tof1-Csm3 complex appears to require an additional input from Mrc1, perhaps to promote the DRC and/or to stabilize forks at the block. Significantly, TIPIN is involved in fork protection from trapped topoisomerase 1 in chicken (DT40) cells (Hosono et al., 2014). Given the high sensitivity of FPC mutants to drugs that trap Top1 and Top2 on DNA, the notion of a primary role of FPC in coordinating topoisomerase and CMG activities warrants further investigation.

Relief of positive supercoiling ahead of the replication fork can also be brought about by rotation of the fork itself (Champoux and Been, 1980), which has the effect of converting supercoils ahead of the helicase into intertwines (also known as catenanes) behind the fork. Fork rotation and catenane formation does not resolve the topological problem, but rather displaces and postpones it for subsequent resolution by topoisomerase type II enzymes (Top2 in yeast). This mechanism of resolving the topological challenge of replication (fork rotation) appears, though, to be limited to sites of replication termination or fork blocks (Keszthelyi et al., 2016). Interestingly, this restriction is imposed by Tof1-Csm3, through an as yet unknown mechanism. Indeed, loss of Tof1-Csm3, but not their Mrc1 partner, leads to elevated post-replicative chromatid entanglement in the absence of Top2 activity (Schalbetter et al., 2015), something that may result from fork rotation.

In addition to parental DNA topology, nascent strand formation at the replication fork could also contribute to sister chromatid intertwining. Indeed, it was suggested that sister chromatid entanglement could result from coupling of leading and lagging polymerases behind the fork (Kurth et al., 2013). This source of entanglement is believed to be resolved in bacteria by transient dissociation of lagging strand polymerase (Kurth et al., 2013). Given the reported role of FPC in regulating polymerase-helicase coupling (Katou et al., 2003) it is tempting to investigate whether events behind the fork could contribute to chromatid catenation in eukaryotes. Baxter and colleagues used RPA recruitment as a proxy for the coupling function of FPC (Westhorpe et al., 2020) but in the absence of a more direct measurement of polymerase-helicase interactions it remains unclear whether RPA enrichment closely reflects replisome coupling. Therefore, as of now it seems unclear whether the precatenanes observed in FPC mutants stem from fork rotation, separate rotation of the polymerases around the parental strands, or from both of these phenomena.

The molecular mechanism of topoisomerase-stimulated pausing is currently unclear. It was speculated to stem from decreased torque at the replisome (and therefore a decreased ability to “pry” the barrier off through rotation about the helix axis) or FPC-dependent CMG inhibition when the replisome encounters topoisomerase(s) in front of a fork (Shyian et al., 2020), possibilities warranting further testing. It will be interesting to use FPC *sof* mutants to untether topoisomerase from the replisome and measure torsion in the vicinity of forks genome-wide, as well as binding of histone and non-histone chromatin components. Indeed, Top1 and Top2 action in the context of transcribing RNA polymerases was shown to prevent nucleosome disruption (Teves and Henikoff, 2014).

Thus, topoisomerases I and II are new players required for replisome pausing at proteinaceous RFBs in yeast. Further studies will clarify the exact mechanism of this unanticipated action and address whether topoisomerases are also involved in pausing in mammals and other metazoan organisms.

### Emerging Replisome Progression Models

The CMG/replisome is a powerful machine capable of rapidly progressing through barriers *in vitro* [(Yeeles et al., 2017; Hizume et al., 2018); reviewed in Hizume and Araki (2019)] but in a cellular context it is “tamed” by the FPC and thus pauses as it approaches stable protein-DNA complexes. As was elegantly revealed in a recent structural study (Baretic et al., 2020), being placed at the front of the fork, between incoming chromatin and CMG helicase, the FPC complex would appear to be in an advantageous position to govern replisome progression in case of encounters with barriers.

**FIGURE 5:**
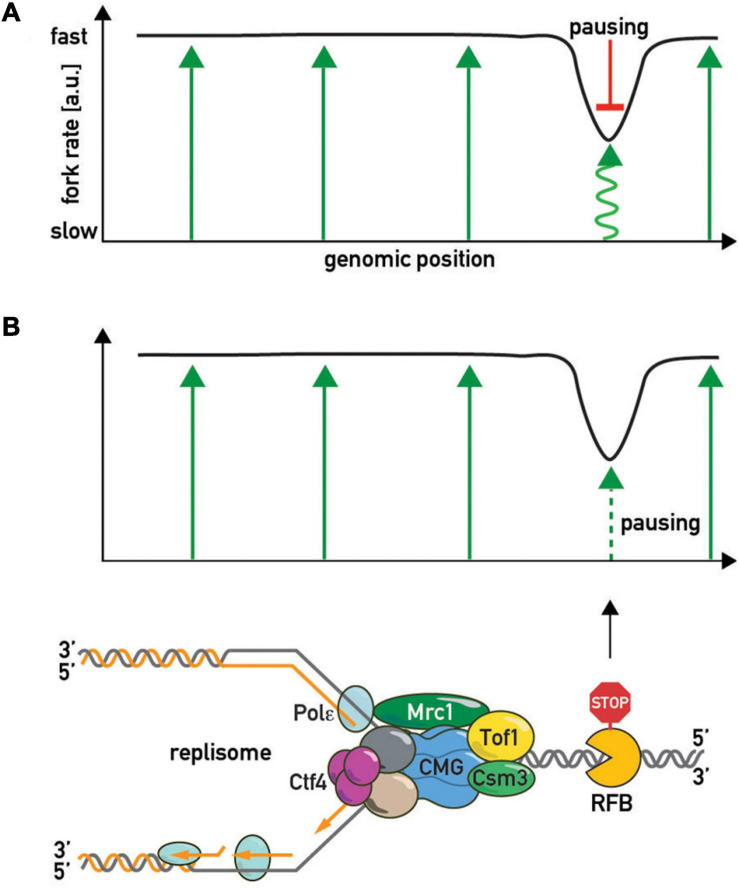
The “slowing-centric” and “acceleration-centric” replisome progression models. Green arrows—fork acceleration. Pausing is either a separate active process with a dominant effect over acceleration [**(A)** red inhibitory bar line] or results from local loss of acceleration function [**(B)** smaller dashed green arrow].

Although the central role of the FPC in fork acceleration and pausing is evident, it is still unclear exactly how it imparts these two apparently opposing effects on the replisome and whether these effects are interconnected. Two models have been postulated to explain how the FPC controls fork rates (Figure 5). In the first (“pausing-centric”) model (Figure 5A), fork acceleration and pausing are unrelated phenomena and the FPC promotes both independently. In this model the FPC globally accelerates forks while its separate pause-promoting activity is triggered locally in the vicinity of a barrier to slow down the replisome. Consistent with this idea, the human FPC complex inhibits CMG activity *in vitro* (Cho et al., 2013). This model is also supported by the observations that the Mrc1/CLASPIN factor has a FPC-shared positive role in acceleration, but does not affect pausing (Hodgson et al., 2007). Thus fork acceleration and fork pausing appear to be separable functions. In the second (“acceleration-centric”) model (Figure 5B), pausing and acceleration are viewed as different sides of the same coin, with pausing simply the result of a local loss of acceleration function at a barrier. According to this model, the FPC accelerates replisome movement everywhere except at RFBs. In other words, the “acceleration-centric” model views pausing as the absence of acceleration. This model is attractive due to its parsimony—there is no need for two separate FPC functions since both effects result from the same ON/OFF acceleration switch. However, since *mrc1*Δ mutants have slow forks but normal pausing (Hodgson et al., 2007), this model would need to invoke an additional Mrc1-independent fork acceleration mechanism, whose existence is not supported by available biochemical data (Yeeles et al., 2017) that are largely interpreted to mean that the FPC simply modulates the dominant effect of Mrc1 on fork rates. Nevertheless, given that there is genetic evidence that FPC and Mrc1 also have non-overlapping roles [i.e., an additive decrease in viability in double mutants (Katou et al., 2003; Shyian et al., 2020)], it is conceivable that the FPC may contribute to acceleration both within a Mrc1 pathway and outside of it. According to the “acceleration-centric” model, the FPC’s general fork acceleration activity would need to be specifically diminished next to a barrier. This could occur either through FPC modification [e.g., phosphorylation (Bastia et al., 2016)], a conformational change, or even transient dissociation from the replisome with potential re-association following RFB bypass (Lewis et al., 2017).

**FIGURE 6:**
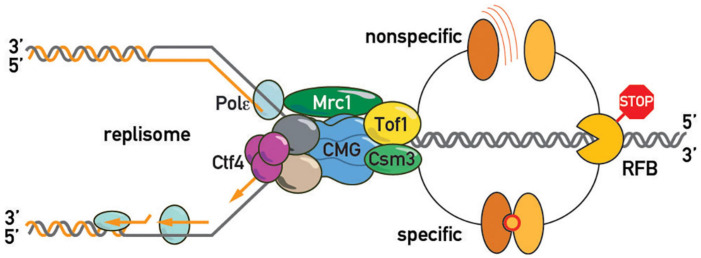
Barrier recognition modes. The replisome recognizes barriers either non-specifically (top of the schematic; by clashing with a roadblock, e.g., tightly bound protein, or an altered DNA topology, e.g., supercoiled DNA, as shown in Figure 4) or through specific protein-protein interactions between barrier and replisome proteins (bottom of the schematic).

Another debatable issue concerning the fork pausing mechanism is whether barrier recognition is either non-specific or utilizes evolved protein-protein barrier-replisome interactions, or whether both mechanisms co-exist in the cell (Figure 6). In the first scenario, pausing is viewed as a general consequence of non-specific replisome encounters with barriers such as high affinity protein-DNA complexes. One might predict then that any tight protein-DNA complex, such as a high-affinity transcription factor or even a nucleosome, will lead to some degree of replisome pausing. Consistent with this view, bacterial transcriptional factors (TetR and LacI) efficiently block eukaryotic replisomes (Hizume et al., 2018). Moreover, a recent study also revealed fork pausing at nucleosomes during DNA replication in a frog egg extract system (Gruszka et al., 2020). On the other hand, the “non-specific recognition” model is challenged by the observation of FPC- and Rrm3-independence of bacterial Tus/Ter barriers when they are “transplanted” into budding yeast (Larsen et al., 2014). However, since Tus/Ter are also able to block helicase-independent mechanical unzipping of the DNA helix they might constitute a unique RFB type (Berghuis et al., 2018). More “RFB-transplantation” experiments are required to confirm the notion that the FPC recognizes only cognate RFBs. If the cell evolved specific surfaces on the FPC unique for each proteinaceous barrier, the specific protein-protein interactions required for pausing might be revealed by screens based on yeast 2-hybrid or protein complementation assays with FPCs from various organisms. These studies might reveal co-evolving FPC-RFB interaction surfaces, if they exist.

In summary, although recent studies have implicated topoisomerases in some form of communication between fork barriers and the FPC, the mechanisms that control the rate of fork movement at barriers are still very poorly defined. As discussed below, new experimental approaches seem necessary to reveal underlying mechanisms.

### Fork Pausing Functions

Although replisome pausing at a number of different fork barrier types (e.g., large protein-DNA complexes, protein-DNA crosslinks, chemically modified bases and alternative DNA structures) has been extensively studied, the physiological role of fork speed regulation at barriers is still poorly understood. The conservation of replisome pausing throughout evolution suggests that it confers a selective advantage, though precisely why and how is often unclear. In the following paragraphs we highlight some of these issues, beginning with examples of “programmed” pausing observed at specialized chromosomal elements such as rDNA repeats, centromeres, telomeres, sites of directed gene conversion linked to mating-type switching, and replication termination sites. We then turn to more general examples of an “accidental” nature, such as transcription/replication collisions or blocks created by covalent modifications to genomic DNA, where links to genome stability are perhaps more apparent.

#### rDNA Recombination, Structure and Stability

The rRFB induces homologous recombination within the rDNA repeat locus and plays an essential role in adjusting the size of the array, either through unequal crossing-over or through the (reversible) generation of extra-chromosomal rDNA circles (Kobayashi et al., 1998). How and why cells sense and regulate rDNA copy number is a fascinating question [reviewed in Kobayashi (2014)]. Current evidence (Iida and Kobayashi, 2019a; Michel et al., 2005) supports a model (Iida and Kobayashi, 2019b) in which expression of Sir2, a known repressor of recombination within the rDNA locus (Gottlieb and Esposito, 1989), is regulated by UAF, a key RNA polymerase I transcription factor, whose availability at the *SIR2* promoter is proposed to vary inversely with rDNA repeat copy number (Iida and Kobayashi, 2019b). One puzzling feature of rDNA copy number regulation is that only about one-half of the normal number of repeats (∼150–200 in most laboratory strains) is transcribed even under optimal growth conditions. The “extra” un-transcribed rDNA copes may allow for sufficient cohesin binding within the rDNA locus to promote recombinational repair of DNA damage there (Ide et al., 2010). Consistent with this notion, Fob1/rRFB-induced recombination may also be important for gene conversion-based correction of mutations within repeat copies (Ganley and Kobayashi, 2007), which are expected to be frequent due to high levels of transcription and fork breakage (Blokhina and Buchwalter, 2020). In summary, replisome pausing at the rDNA locus in budding yeast is a highly regulated process that appears to have evolved to help meet the unique demands of this heavily transcribed and repetitive region of the genome.

#### Kinetochore Assembly and Function

Even the small (∼100 bp) “point” centromeres of budding yeast nucleate the assembly of a large kinetochore complex, involving extensive DNA looping of ∼25 kbp of flanking “pericentric” sequences (Yeh et al., 2008). As pointed out above, centromeres in budding yeast are prominent sites of fork pausing. Notably, both Csm3 and Rrm3 have been implicated genetically in *de novo* kinetochore assembly, in a manner which suggests that slowing down replication fork progression directly promotes this process (Cook et al., 2018). One model proposes that reduced fork velocity at centromeres acts by favoring loop formation in pericentric regions (Lawrimore and Bloom, 2019). Although molecular details are yet to be worked out, emerging evidence links replication fork pausing to condensin- and cohesin-mediated loop formation not just at centromeres, but also within the rDNA, which share several other common features [reviewed in Lawrimore and Bloom (2019)].

#### An Epigenetic Imprint Controlling Mating-Type Switching

Unidirectional replication due to a specific pausing site is essential for an imprint placed in a strand-specific manner at the *mat* locus in fission yeast. This epigenetic mark in some way directs a gene conversion event required for correct mating-type switching pedigree and is thus one of the clearest examples of a specialized physiological process that utilizes fork pausing as part of its molecular mechanism of action. Although the exact nature of this imprint is still unclear, it would appear to consist of an alkali-labile nucleic acid component or modification, perhaps a ribonucleotide (Raimondi et al., 2018).

#### Replication Termination

In bacteria, the well characterized Ter/Tus system controls replication termination by trapping the two convergent replication forks within a defined region of the genome [reviewed in Dewar and Walter (2017)]. Studies in budding yeast suggest that regions where opposing replication forks converge are also enriched for pausing elements (Fachinetti et al., 2010). A challenge for future studies will be to determine if and how fork pausing plays a role in completion of replication at converging forks and resolution of sister chromatids.

#### Telomere Replication and Telomere Repeat Length

Originally described in budding yeast (Ivessa et al., 2002; Makovets et al., 2004), but more recently characterized in fission yeast and mammalian cells (Miller et al., 2006; Sfeir et al., 2009), fork progression is decreased or blocked in the vicinity of telomeric repeat sequences. This block may be the direct result of replisome interference by G-quadruplex structures formed by telomere repeats, since it is exacerbated by mutations in helicases known to unwind such structures (Crabbe et al., 2004; Ding et al., 2004; Sfeir et al., 2009). Although one might imagine that proteins binding tightly to telomere repeat sequences, such as Taz1 in fission yeast or TRF1 in mammalian cells, would inhibit fork progression at telomeres, both proteins have instead been shown to do just the opposite (Miller et al., 2006; Sfeir et al., 2009). Whether the same is true for the budding yeast telomere repeat binding protein Rap1 has not yet been tested.

#### Preventing Replication-Transcription Collisions

The first pausing site to be identified, the rDNA RFB, was proposed to have evolved to prevent (or at least reduce) collisions between the replisome and transcription complexes that might lead to DSBs and consequent genome instability (Brewer and Fangman, 1988). This idea was based upon the high rDNA transcription rate and the polar nature of the RFB, which, together with the proximity of the rDNA replication origin to the barrier, means that most replication occurs in the same direction as RNA polymerase I transcription. However, *fob1*Δ cells with a full rDNA repeat locus do not display overt evidence of transcription-dependent replisome collisions (Takeuchi et al., 2003). Nevertheless, reduction of rDNA repeat number from ∼150 to 20 does lead to a measurable level of transcription-induced fork arrest in *fob1*Δ cells (Takeuchi et al., 2003), suggesting that unusually high levels of rDNA transcription can lead to replisome collisions. Thus, to what extent and under which conditions the Fob1-RFB in budding yeast (and similar rRFBs in other organisms) protects against replication-transcription collisions and contributes to cell fitness is still an open question. The answers to this and other questions may emerge from genetic approaches that can systematically explore the vulnerabilities of *fob1* mutants, such as synthetic genetic array [SGA; (Tong et al., 2001)] or transposon saturation [e.g., SATAY (Michel et al., 2017)] screens. One attractive genetic background for these screens will be *mre11*Δ due to its exquisite sensitivity to Fob1 protein levels (Bentsen et al., 2013).

#### Pausing May Help in Navigating Covalently Linked Protein Barriers to Promote Fork Continuity

During catalytic cycles topoisomerases transiently connect to DNA *via* covalent bonds. It is documented that high levels of Top1 are stably and covalently attached at Fob1 rDNA barriers (Di Felice et al., 2005; Krawczyk et al., 2014), independently of transcription and replication (Di Felice et al., 2005). Given the replisome interaction with topoisomerases and the important role of the FPC in protecting cells from these trapped enzymes (Redon et al., 2006; Reid et al., 2011; Shyian et al., 2020), it is tempting to propose that pausing may help to prevent fork collision and collapse at these sites (Strumberg et al., 2000). It will be important to investigate whether topoisomerases also highly accumulate at other barriers throughout the genome and whether the FPC has a general protective role at all of these locations. Similarly, it will be interesting to test whether the FPC may also promote pausing at other covalently linked proteins ahead of the fork (Stingele et al., 2017), especially given the proposed Tof1 interaction with Wss1 protease (O’Neill et al., 2004), which degrades proteins cross-linked to DNA.

#### Does the FPC Act Ubiquitously at Tightly Bound Proteins and Protein Complexes?

Although some DNA-binding proteins (e.g., Fob1 or Tus in bacteria) may promote fork arrest through mechanisms independent of their DNA-binding affinity, it seems likely that strong DNA binding in and of itself can cause fork blockage. Indeed, the most effective blocks so far characterized consist of arrays of binding sites for high-affinity DNA-binding proteins, such as yeast Rap1 or the bacterial LacI repressor (Goto et al., 2015). However, even single sites for strongly bound complexes (e.g., ORC, TFIIIB, and perhaps tight-binding pioneer transcription factors) may require a replisome pausing mechanism mediated by the FPC to reduce the risk of fork collapse following collisions. Although still speculative, FPC action may also serve to promote factor re-binding following fork passage and/or to facilitate histone inheritance pathways [reviewed in Rowlands et al. (2017)].

#### Replisome Pausing May Contribute to Polymerase-Helicase Coupling

Although often thought of as a stably linked and highly coordinated complex, the DNA polymerases and helicase at the replisome may operate in a highly independent manner that requires an inherent mechanism to avoid excessive uncoupling of the two machines (Katou et al., 2003; Pacek et al., 2006; Graham et al., 2017). Upon depletion of dNTPs or encounters of DNA adducts, replicative DNA polymerases would slow down and lag behind the CMG, if not for a connection (“coupling”) between the two machineries. Mutation of FPC components or *MRC1* lead to separation of nascent DNA signal (BrdU) and CMG helicase components in cells challenged with HU (Katou et al., 2003). The exposed ssDNA between helicase and polymerase is covered with RPA, which strongly accumulates ahead of the polymerase in FPC and *mrc1* mutants (Westhorpe et al., 2020). Mrc1 interacts with polymerase epsilon (Lou et al., 2008), and with CMG and FPC (Baretic et al., 2020), suggesting direct protein-protein coupling. Moreover, recent structure-function dissection of the FPC component Tof1 showed that pausing and replisome coupling functions are tightly linked (Westhorpe et al., 2020). Thus, FPC-mediated fork slowing may serve a role here as a means to couple replicative helicase and polymerase, thereby decreasing ssDNA buildup at forks and potentially preventing global RPA exhaustion.

#### The Perils of Excessive Pausing

Although the FPC would appear to have adaptive functions with respect to genome stability in most contexts, its dysregulation or action in certain mutant backgrounds can actually be deleterious. For example, excess pausing activity, which might seriously delay replication, could lead to genome instability, through under-replication and subsequent damage (e.g., DNA bridges leading to DSBs) during mitosis (Mohebi et al., 2015; Ait Saada et al., 2017). In yeast, FPC action is actually deleterious in MRX-deficient cells experiencing additional replication difficulties (Shyian et al., 2016, 2020) and in cells with a compromised Smc5/6 complex, which is proposed to be involved in DNA damage tolerance (Menolfi et al., 2015). Furthermore, a recent study showed that both Claspin and Timeless expression are increased in primary tumor samples and act to increase replication stress tolerance in these cells through their direct action on replication forks (Bianco et al., 2019; Pasero and Tourriere, 2019).

Thus replication pausing, and the FPC as its main executive, carries out many different roles in yeast cells. It is worth noting here that the essential role of the FPC in pausing makes it tempting to study the cellular consequences of pausing loss by simply inactivating one or both of its components. However, interpretation of observations in FPC null mutants may be confounded by the multiple additional roles of the FPC in checkpoint, fork rotation, and sister chromatid cohesion functions (McFarlane et al., 2010; Hizume and Araki, 2019). Using the recently described FPC *sof* mutants specifically deficient in pausing but proficient in other functions (Shyian et al., 2020; Westhorpe et al., 2020) will be crucial to place the spotlight on pausing by retaining other roles intact.

### Perspectives

#### Structural Elucidation of the Replisome in Different Functional States

Given recent advances in cryoEM-derived structures of replisome complexes (Eickhoff et al., 2019; Baretic et al., 2020; Kose et al., 2020; Rzechorzek et al., 2020; Yuan et al., 2020), we anticipate further accumulation of structures of even more complex replisome assemblies. In particular, structural comparison of a normal elongating replisome with those stalled at specific RFBs will help to address the question of barrier-specific versus non-specific recognition by revealing protein-protein interfaces in front of the helicase. Moreover, some parts of the FPC and most of Mrc1 were not resolved in the most recently published replisome structure (Baretic et al., 2020). Future studies may yield valuable new information.

#### Molecular Mechanisms of Pausing

Recent *in vitro* reconstitution experiments defined a minimal set of proteins required to elicit pausing at linear non-chromatinized DNA substrates (Hizume et al., 2018; Baretic et al., 2020). Given the likelihood that DNA topology plays an important role in pausing, expanding these studies through the use of closed circular DNA templates, where topology can be quantified and manipulated, could reveal the causal relationship between topology and pausing at RFBs. Single-molecule approaches allowing for controlled application of torsional stress may be particularly informative. Further *in vitro* studies, either in bulk solution or at the single-molecule level, are likely to explore the role of additional replisome-associated factors, post-translational modifications, and nucleosomes.

#### Comprehensive RFB List

Genome-wide screens in FPC *sof* mutants will help to clarify the physiological roles of pausing and barriers. To address understudied barriers, we anticipate the development of systems to stall replication forks directly *via* a transcribing RNA polymerase complex [as opposed to blockage by the pre-initiation complex at promoters or by R-loops behind a RNAP; (Gomez-Gonzalez and Aguilera, 2019)] to investigate the consequences of head-on versus co-directional collisions. Refinements of available methods and development of novel approaches to investigate *in vivo* fork progression at high resolution will help to identify more subtle irregularities in fork rates that could nevertheless have important functional consequences (Gruszka et al., 2020). Such studies might reveal, for example, whether forks pause *in vivo* at enhancer- or promoter-bound TFs, or at nucleosomes, and if so, whether there are functional consequences.

#### Harnessing FPC Biology

Given TIMELESS-TIPIN’s pro-oncogenic role (Bianco et al., 2019; Pasero and Tourriere, 2019) and degradation of either component in the absence of its partner (Chou and Elledge, 2006; Bando et al., 2009) the interaction interface of the FPC constitutes an attractive druggable target. Chemogenomic screens for TIMELESS-TIPIN degradation may identify compounds inducing degradation of the FPC, thus killing cancer cells.

### Conclusion

Discovered more than three decades ago, replication fork pausing still poses many unresolved questions as to mechanisms and physiological roles. However, as new approaches to measure pausing are devised, additional pausing factors identified, regulated systems engineered and recombinant minimal systems reconstituted, the field advances. Topoisomerases were recently found amongst the positive regulators of pausing, which establishes a novel link between replisome progression and topological transitions at the fork. The relation between torsional stress and chromatin resistance to replisome progression will be an important venue for future research.

## Author Contributions

MS and DS reviewed literature, designed sections of the manuscript, and wrote and revised first draft. MS made figures. Both authors contributed to the article and approved the submitted version.

## Conflict of Interest

The authors declare that the research was conducted in the absence of any commercial or financial relationships that could be construed as a potential conflict of interest.

## Abbreviations

FPC: Fork Pausing Complex (ScTof1-Csm3; SpSwi1-3; HsTIMELESS-TIPIN)
RF: Replication Fork
RFB: Replication Fork Barrier
*sof*: separation-of-function mutation
MTC: Mrc1-Tof1-Csm3 complex
Topo I, Topo II: Topoisomerases I and II
DRC: DNA Replication Checkpoint.
